# An Assessment of Outpatient Satisfaction with Hospital Pharmacy Quality and Influential Factors in the Context of the COVID-19 Pandemic

**DOI:** 10.3390/healthcare10101945

**Published:** 2022-10-05

**Authors:** Binh Quoc Nguyen, Cuc Thi Thu Nguyen

**Affiliations:** 1 Pharmacy Department, Cho Ray Hospital, Ho Chi Minh City 700000, Vietnam; 2 Faculty of Pharmaceutical Management and Economics, Hanoi University of Pharmacy, Hanoi City 100000, Vietnam

**Keywords:** drug dispensing, patient satisfaction, pharmacy service, SERVQUAL, Vietnam

## Abstract

The study aims to evaluate outpatient satisfaction (OS) with health insurance drug dispensing at the central hospital in Vietnam and to explore the influential factors. A cross-sectional survey was conducted on adult outpatients via an adjusted SERVQUAL questionnaire. The questionnaire’s internal consistency (Cronbach alpha) and construct validity (exploratory factor analysis) were considered. The difference between groups was solved using a *t*-test or ANOVA-test. The multiple-regression analysis determined the influence levels of each factor. A *p*-value less than 0.05 was statistically significant. A total of 210 participants participated, with most being over 55 years old, female, with a high school education, and freelancers. The mean general satisfaction score was 3.42 (SD = 0.79). The reliability obtained the highest satisfaction score, and the guarantee was the lowest. The final questionnaire, including five factors (reliability, responsiveness, assurance, sympathy, and tangible) with 26 observational variables, had an internal consistency reliability and construct validity. These five factors had a statistically significant correlation and influence on the general satisfaction of the outpatients. The reliability factor had the strongest influence, and assurance had the weakest. Training staff about communication, counseling, and consolidating the facilities are the core solutions for increasing OS.

## 1. Introduction

Healthcare services of a high quality are one of the Sustainable Development Goals that need to be achieved and are a top concern of many nations [1]. Although there are many different approaches to ensuring quality health services, they need to be effective, safe, and patient-centered [2,3]. In other words, these services should be evidence-based; avoid causing danger to patients; and respond to individual preferences, needs, and values [4]. Therefore, patient satisfaction surveys are the first core steps to building and assessing medical care systems in many countries, in order to explore the outstanding problems in patient health examinations and treatments, thereby indirectly enhancing the compliance of patients, and increasing revenue and the reputation of medical facilities and physicians in the health market [5].

Vietnam has three levels of the public healthcare system (central, provincial, and district levels) and offers universal health insurance coverage [6,7]. In addition, Vietnam is experiencing an aging population, with the estimated number of people aged 65 years and over expected to increase to 28% in 2050 [8]. This leads to high pressure on the public health system, especially at the stage of drug dispensing. In Vietnam, health insurance co-payments are only implemented in hospitals, which means that health-insurance drugs are only provided by hospital pharmacies [9]. As the last step in outpatient medical examination and treatment, health insurance dispensing may create negative reactions in patients due to previously accumulated fatigue and displeasure. However, satisfying patients is also a massive challenge for hospital pharmacy staff. Because of a low ratio of healthcare workers per capita in Vietnam [10], pharmacists at the hospital have a lot of work pressure, which comprises guaranteeing good dispensing practice for drugs, monitoring adverse drug reactions, managing drug storage, reviewing medication utilization, drug compounding, and other administrative procedures. These workloads are even more significant in central hospitals with a great number of patients, because of a tendency to prefer using medical services at higher levels [11]. In addition, in the COVID-19 pandemic context, some characteristics of the health service system were different before the pandemic [12], which may affect patient satisfaction. Specifically, in Vietnam, the process of the examination and treatment, and for receiving drugs is controlled strictly; patients require more complicated procedures to prevent the spread of the pandemic (health declaration, COVID-19 screening test, wearing medical masks, using alcohol-based hand sanitizer, etc.). Hospital pharmacies have been facing increased difficulties in human resources for dispensing and counseling drugs because of the compulsory participation in anti-pandemic activities and the high risk of drug shortages due to difficulties regarding transportation and supply disruption [13,14].

Among the central hospitals in Vietnam, Cho Ray Hospital is the leading general hospital and is the last line in the medical examination and treatment system of Southern Vietnam. The hospital has 2300 beds. The average inpatients per day is 2544 and for the outpatients it is 3500; more than 65% of hospital visits are outpatients. Moreover, the hospital is ranked as an advanced special hospital thanks to the constant improvement in service quality, which results in a significant contribution to healthcare for the people of the southern region, as well as the whole country and foreigners. Therefore, a great concern for hospital leaders is ensuring patient satisfaction with dispensing drugs, identifying the factors that make patients unsatisfied, and suggesting appropriate recommendations to improve the quality of health insurance drug dispensing. Especially in the difficult period of the COVID-19 pandemic, it is crucial to ensure safety, sufficient drugs, and human resources in order to serve patients.

As far as we know from the literature through preliminary searching the Pubmed database, there have been limited studies in Asia [15,16,17], and most have been conducted in Ethiopia [18,19,20,21,22,23,24,25,26]. According to the studies conducted in Ethiopia, nearly half of the patients were unsatisfied with the pharmaceutical care services in the specified hospitals [18,20], and the uncomfortable private counseling areas and waiting areas were negatively associated with patient satisfaction [19]. Moreover, there are not many studies that have used the SERVQUAL instrument in order to evaluate the performance of pharmaceutical services [27]. Most papers evaluating the patient perceptions of pharmacy service used questionnaires that they designed themselves [15,16,19,22,24,25]. The studies using SERVQUAL in medical services focused on physician performance or hospital performance, not on pharmacy services [27,28,29,30].

This study aimed to assess outpatient satisfaction with health insurance drug dispensing at the central hospital, and to determine the influential factors the levels they affected outpatient satisfaction. The findings explored the most challenging period of the COVID-19 pandemic, and can be used to orient policymakers regarding appropriate and effective interventions in order to enhance patient satisfaction.

## 2. Methods

### 2.1. Study Design and Subjects

Our study comprised a combination of qualitative research to modify and develop a questionnaire, and quantitative research to evaluate outpatient satisfaction and explore the influential factors. We selected outpatients who were 18 years or older receiving health-insurance drugs. The patients or one representative member in their family were invited to a face-to-face interview when they came out of the pharmacy dispensing counters. Disabled people, foreigners, and patients with acute symptoms were excluded.

This study was approved by the Institutional Review Board, no. 4401/QĐ-BVCR, dated 25 October 2020, and was conducted from January to April 2021 at Cho Ray Hospital Vietnam. The interviewees were informed of the research information and written consent was required before entering the face-to-face interview. Participation was voluntary, and respondents could withdraw at any time during the interview. Patient information and interview content were coded and kept confidential.

### 2.2. Development of the Outpatient Satisfaction Evaluation Questionnaire

Items evaluating outpatient satisfaction were initially based on the SERVQUAL questionnaire in a prior study [31], and were modified according to the study situation of the hospital pharmacy service. The SERVQUAL method is used to assess service quality based on standardized parameters and consists of five domains of service quality attributes, including tangibles, reliability, responsiveness, assurance, and empathy [27]. Reliability is defined as the organization of correctly performing services from the first visit; assurance is the staff’s courtesy and knowledge, as well as their capacity to make the clients feel confident and boost trust; responsiveness involves the willingness of staff to fulfill customers promptly, promote services, and respond to service inquiries; tangibles are the physical facilities; and empathy means caring, paying attention to customers, and providing services to customers [27,32].

A pre-test was carried out to check the interviewees’ understanding of the contents, the terminology, and the words used in the questionnaire, and to make further modifications. The pre-test was conducted with 16 in-depth interviews, comprising 5 pharmacists who dispensed outpatient insurance drugs and 11 outpatients or their caregivers. The in-depth interviews, in combination with the criteria of the Ministry of Health for evaluating the quality of Vietnamese hospitals and the government’s regulations on organization and pharmacy activities of medical examination and treatment facilities [33,34], resulted in 29 observational variables, with four variables for reliability, five for responsiveness, seven for assurance, four for empathy, eight for tangibles, and one for the patient overall satisfaction (Table 1). Each question was responded to on a five-point Likert scale ranging from 1 (not satisfied) to 5 (very satisfied).

The mean score for each item or domain was estimated by dividing the total satisfaction score and the total number of study participants. Then, the average score was evaluated and classified on the following interval scale: dissatisfied (less than 2.61), average (between 2.61 and 3.4), satisfied (between 3.41 and 4.2), and very satisfied (over 4.2).

### 2.3. Sampling Method

The sample size was calculated based on the recommendation of Hair et al. [35], whereby the minimum sample size was the number of indicator variables and estimated parameters. For all 29 observational variables and the five-point Linkert scale, the minimum sample size in the research was 145 respondents. The technique of convenient sampling was applied in this study.

The hospital has about 1400 health-insurance-coverage patients out of the 3500 outpatients seen per day. The patients had a follow-up visit within one month. Therefore, face-to-face interviews would be conducted daily (except for Sunday) and within two weeks, with about 18–20 patients interviewed per day.

### 2.4. Statistical Analysis

The reliability of the questionnaire’s internal consistency was estimated using Cronbach’s alpha and corrected item–total correlation. Cronbach’s alpha value over 0.6 and corrected item–total correlation over 0.4 determined a good internal consistency for all items of each domain [35,36,37].

Exploratory factor analysis (EFA) was used to assess the validity of the questionnaire construct and that variable selection was maintained in the questionnaire. The factors were extracted by principal component analysis, with the following criteria a Kairer–Meyer–Olkin of at least 0.5, Bartlett test with a *p*-value less than 0.05, an Eigenvalue over 1.0, and total variance explained higher than 50% [35]. Moreover, reclassifying the measure items to increase the interpretability of these factors was solved by Varimax with Kaiser Normalization and principal component analysis. As a result, the cut-off point for the factor loadings and the difference in factor loading between the highest value and the value of each item was at least 0.5. 

The difference between groups was solved by *t*-test or ANOVA according to the type of variables. The multiple-regression analysis determined the influencing levels of each factor on the outpatient satisfaction with insurance-drug dispensing. A *p*-value less than 0.05 was set as being statistically significant. All of the analyses were performed using R version 4.1.0 (R Core Team, Vienna, Austria).

## 3. Results

### 3.1. Characteristics of Respondents

A total of 210 out of 218 survey sheets meeting the inclusive criteria were obtained. Most of the patients were over 55 years old, female, with a high school education, and freelancers (Table 2).

### 3.2. The Mean Score of Items

Table 3 shows that outpatients were satisfied with Cho Ray hospital’s health insurance drug dispensing service, with a mean overall satisfaction score of 3.42 (SD = 0.79).

Among the five domains of SEVQUAL, the outpatients indicated reliability with the provision of health-insurance drugs (mean overall score of 3.85, SD= 0.62), in which the highest score was found for quality-assured drugs (mean score of 4.26, SD = 0.75). 

The second domain with the high score was tangibility, with a mean overall score of 3.69 (SD = 0.54). Out of these items, the highest score was the available equipment for COVID-19 prevention in the facility (mean overall score of 3.98; SD = 0.81), and the lowest was cleanliness (mean overall score of 3.39; SD = 0.95). 

Third, sympathy had a mean score of 3.39 (SD = 0.70). Patients were the most satisfied with the pharmacy staff’s care for each person, but the least satisfied with their listening to patient issues. Regarding responsiveness and assurance, the participants assessed the service capacity as average (mean score of 3.35 (SD = 0.55) and 3.33 (SD = 0.79), respectively). The lowest-ranked aspects of these domains were the reasonable cost of each prescription (mean score of 3.18, SD = 18) and the careful listening of the staff to customer requests (mean score of 3.14, SD = 1.12), respectively. 

### 3.3. Reliability and Validity of the Scale

Considering the scale’s reliability for the test taken for the first time, as shown in Table 3, reliability REL1 and tangibles T1 were omitted because they had a corrected item–total correlation less than 0.5, and the Cronbach’s alpha was deleted as it was greater than the total allowable Cronbach’s alpha. The re-run test ensured the reliability of the scales, with the results of the total Cronbach’s alpha for all factors above 0.7 and a corrected item–total correlation of over 0.5.

After excluding the variables REL1 and T1, the final 26 observational variables were evaluated using EFA with principal component factoring analysis and Varimax rotation, with the following results: (1) the EFA was completely consistent (KMO value = 0.847), (2) the observational variables were correlated with each other (*p*-value of Bartlett’s test < 0.001), (3) the factors had a good summary information meaning (Eigenvalue = 1262), (4) factors explained more than 62% of the variation of data (total variance extracted = 62.89%), and (5) items had factor loadings > 0.5. Therefore, these five factors were appropriate and valid, which meant that the research scale did not need to be readjusted (Table 4).

### 3.4. Multiple-Regression Analysis

The Pearson correlation analysis in Table 5 indicates that all factors had a statistically significant correlation with the dependent variable (general satisfaction), with r > 0 and *p*-value < 0.05.

The results of the regression model analysis assessing the levels of the five influential factors on the general satisfaction variable are shown in Table 6. 

The multiple model explained 63% of the variation for the dependent variables (general satisfaction) (adjusted-R^2^ coefficient of 0.631) and had no autocorrelation (value of the Durbin–Watson test of less than two). There was a statistically significant consistency in the regression model with the included variables (*p*-value of ANOVA test less than 0.001). The independent variables were the significant explanation of outpatient general satisfaction (*p*-value < 0.05). There was no multicollinearity (VIF coefficient < 2), and all independent variables had the same effect on the dependent variable (regression coefficients > 0). The reliable factor had the strongest influence on general satisfaction, and assurance was the weakest. The regression equation had the following form:

GS = 0.464 × REL + 0.287 × T + 0.079 × RES + 0.075 × S + 0.014 × A

## 4. Discussion

Considering hospital pharmacies, patient-centered care has expanded beyond the dispensing role, to directly providing medication instructions and education to patients in order to make sure that the patient can safely self-administer the drug at home, and that they adhere to the treatment [38]. Patient-centered care needs close collaboration between patients and healthcare providers, where health policy decisions are based on the patients’ preferences, needs, and values [38,39]. One of the critical factors in healthcare with a patient-centered approach is patient satisfaction, which is also an indicator that drives improvement in healthcare quality [1,40]. The problem of patients not being satisfied with their examination and treatment at the hospital was identified through a patient satisfaction assessment. Since then, the quality of care has been improved systematically and centrally, gradually meeting customers’ satisfaction and expectations. 

The research results here show that all five components affected outpatient satisfaction with the health insurance drug dispensing service. Each factor had a different impact on satisfaction; specifically, reliability had the strongest influence on outpatient satisfaction with pharmacy services. Patients reported feeling secure with the correct provision of the hospital pharmacists as the prescription with a clear origin and quality assurance, which leads to high satisfaction of outpatients (mean scores of 3.51, 3.53, and 4.25, respectively). Checking prescriptions and comparing the prescribed medication with the correct drug name on the dispensing label are essential strategies to reduce dispensing errors. Therefore, all medical staff at hospital must be trained and enhanced their professional knowledge frequently, as well as practice their skills and comply with the regulations on medical ethics before they are employed to work at the hospital. However, patient were the most unsatisfied with the reliability variable of drug price not being clearly shown on the receipt. Trust was found to be the factor with the highest degree of influence on patient satisfaction and health outcomes [41], so solving problems that have not yet brought satisfaction to patients should be focused on urgently. Therefore, the list of drug prices should be made public and transparent for clients in order to increase patient satisfaction.

The tangibles factor was the second strongest factor affecting outpatient satisfaction, with quite a high score (3.6, SD = 0.7). The patients were satisfied with the hospital’s well-functioning drug dispensing equipment and spacious and airy waiting area for drug dispensing. The comfortability of the waiting area and payment status had a positive association with satisfaction [19,42]. Cho Ray Hospital has dealt with this well and will use it as a basis for maintenance and improvement in the future. In addition, patients had high satisfaction with the drug dispensing area being fully equipped with COVID-19 prevention equipment (mean score of 3.98, SD = 0.81), which might have helped to increase the mean score of the tangibles factor. However, patients were not satisfied with the uncleanness of the medicine dispensing area, which caused discomfort to patients and their family members. Although the pharmacy always has a cleaning team, the large number of patients along with the low frequency of cleaning may make patients feel as though the areas are not always clean. As a result, the hospital should have solutions to overcome this situation, namely, regular cleaning every two hours and checking the sanitary status of the drug supply area. In general, these findings suggest the importance of tangibles elements as patient satisfaction predictors.

Responsiveness was the third most potent factor affecting outpatient satisfaction with health-insurance-drug dispensing services at Cho Ray Hospital. Patients were satisfied with the range of drugs available according to their treatment needs, although there were difficulties relating to human resources and the high risk of drug shortages in the COVID-19 context. This can be explained by the efforts of the hospital pharmacy staff in regularly checking and updating the drug list covered by health insurance in order to adapt to the context. In addition, the hospital pharmacy staff should always strengthen appropriate procurement and proper inventory management so as to guarantee the availability of essential drugs. These are key factors that help ensure an adequate supply of the drug [14,43]. In the responsiveness domain, outpatients were unsatisfied with the waiting time. Besides waiting for a long time, in Vietnam, the waiting room is where medicine is received in central hospitals; this may be one of the reasons that patients feel quite tired. A project in Jordan showed significant results when improving the waiting time for prescriptions and, in turn, increased patient satisfaction after the full implementation of the project [44]. The application of lean management in an outpatient pharmacy was the key driver for effective improvement when reducing patient waiting time and enhancing the satisfaction of both patients and employees [44]. Moreover, the pharmacy department should optimize each stage by applying quality improvement models, arranging and supplementing helpful human resources during peak hours, and opening more drug delivery counters to ensure the need is met during peak hours. The high drug cost is another factor outpatients were unsatisfied with. In Vietnam, despite the universal insurance coverage and the recent policy of 100% reimbursement, out-of-pocket payments from patients are still available for drugs that health insurance does not cover or that it partially covers [45,46]. Therefore, in cases of out-of-pocket payment, patients have the choice to buy from hospital pharmacies or private pharmacies. Although the drug price is issued by the government, this is only a maximum price, which leads to price competition between pharmacies. Therefore, the drug price is still a factor that patients care about and compare. If the hospital pharmacies do not have the best price for the patients, it will result in a loss of revenue for the hospital. Therefore, pharmacists play a vital role in substantial healthcare savings [47].

Regarding the empathy factor, our research illustrated that patients felt satisfied with pharmacists’ respect and care for patient health, but unsatisfied with the staff’s listening and sharing of information with the patient. Therefore, patient overall satisfaction with this factor was not high. The listening and sharing of information by medical staff had a potent effect on patient satisfaction, even higher than kindness and consideration, because outpatients would leave the hospital just after completing the examination and treatment, which means they had fewer opportunities to communicate with medical employees than the inpatients did [48]. In addition, pharmacy staff might have tired facial expressions when working with intense stress and receiving many patients, which might make patients feel that they do not care about their health. In order to increase patient satisfaction, additional training for staff on skills and body language in communication and genuine care is needed; the pharmacy staff should show more empathy for outpatients so as to encouraged revisits, especially with the current intense competition.

Last, but not least, assurance had the weakest impact, but it also contributed to outpatient satisfaction. Although patients felt most satisfied with the support of the pharmacy staff in answering patient questions, they were also not satisfied with the careful listening of the pharmacy staff to their customer requests. The lack of careful listening from medical staff is a common problem in large hospitals when the number of patients is too large, and pharmacists do not have enough time to listen to each customer. However, the availability and active listening from medical staff were among the least satisfactory and most essential items of medical care [49,50,51]. Therefore, our study suggests that the hospital could arrange reasonable human resources during peak hours and open more client care counters or mobile lines in order to have more time to listen to patient requests.

In a meta-analysis published in 2022 [27] that reviewed studies using the SERVQUAL tool for assessing the quality of the service provided, a total of 15 studies were eligible for analysis. Among these, most of the studies were from Iran (53%), only one study was from Southeast Asia, and no studies considered the aspect of the hospital pharmacy service. As a result, the comprehensive development of the research instrument (the SERVQUAL questionnaire with the final 26 observational belonging to five factors) will help to expand similar studies in other hospitals and make a convenient comparison between different patient groups across various geographical areas. The quality of services provided by the different facilities could be improved based on the results obtained from the SERVQUAL tool [27]. Although the SERVQUAL tool has many advantages, its results often showed that the patient’s expectations regarding the service quality were significantly higher than the quality they received [27]. However, high quality is not always guaranteed at all healthcare facilities. It depends on the capacities of the facilities, so it is more important to recognize the factors that need to be improved from the patient’s perspective.

Our research findings contribute to a better understanding of the pharmacy dispensing service quality and the influential factors of patient satisfaction, especially in the leading central hospital with a considerable number of patients. These findings form the basis for strategies in order to improve the current status of patient-centered pharmacy service in medical facilities.

### Study Limitation

We are aware that there are limitations to this study. The convenient strategy of selecting outpatients and the limited data in one hospital decreased the diversity and the representativeness of our study. In addition, our study has limitations related to data collection regarding patient health and drug usage, and there was no sub-group analysis relating to patient characteristics (type of respondents, the patient or the patients’ representative, the age group, gender, occupation, or their levels of education). Demographic factors analysis is necessary, but the results do not provide many good solutions, because we cannot serve a specific group of patients when they feel dissatisfied. Therefore, we paid much more attention to the five domains of service quality attributes, including tangibles, reliability, responsiveness, assurance, and empathy, than the demographic characteristics. The explored issues would form the basis for suggesting potential appropriate and practical solutions in Vietnam going forward, in order to enhance patient satisfaction. This was the objective of this study.

## 5. Conclusions

The outpatient satisfaction with pharmacy dispensing covered by health insurance at the central hospital had an average score. The SERVQUAL questionnaire, which was built with the final 26 observational variables belonging to five factors, was reliable and valid for evaluating outpatient satisfaction. These five service quality components (reliability, responsiveness, assurance, sympathy, and tangibles) had a statistically significant correlation and influence on outpatient satisfaction. The reliable factor had the strongest influence, and assurance was the weakness. The core solutions to increase outpatient satisfaction are strengthening the training for drug dispensing staff regarding communication and drug counseling for patients, consolidating the facilities, and guaranteeing drug supply.

## Figures and Tables

**Table 1:** The contents of the questionnaire.

No	Code	Contents
**Reliability (REL)**		
1	REL1	Drugs are provided accurately as prescribed
2	REL2	Drugs are provided with a clear origin
3	REL3	Drugs are provided with quality assurance
4	REL4	Drug prices are publicly listed, clearly shown on the drug receipt
**Responsiveness (RES)**		
5	RES1	Hospital pharmacy service provides meet the number of types of drugs according to treatment needs
6	RES2	The hospital pharmacy service meets the quantities of drugs according to treatment needs
7	RES3	The cost of a prescription meets the patient’s financial ability.
8	RES4	Operating time to meet the needs of the patient
9	RES5	The waiting time to receive the drug is reasonable
**Assurance (A)**		
10	A1	The staff of hospital pharmacy service support answers patients’ questions
11	A2	Pharmacists guide the use of drugs completely and accurately
12	A3	Hospital pharmacy service had professional pharmacist, drug usage monitoring, drug consultants
13	A4	The staff of hospital pharmacy service always checks carefully before delivering medicine to patients
14	A5	There are rarely errors in health insurance drug dispensing by a staff of hospital pharmacy service
15	A6	Patients can buy drugs outside the health insurance list at the hospital pharmacy
16	A7	The staff of hospital pharmacy service always listen carefully to the requirements of customers
**Sympathy (S)**		
17	S1	The staff of the hospital pharmacy service takes care of each patient
18	S2	The staff of hospital pharmacy service respect patients
19	S3	The staff of hospital pharmacy service does not discriminate between customers when serving
20	S4	The staff of the hospital pharmacy service always listens and sympathies with patients
**Tangibles (T)**		
21	T1	The drug dispensing area is always clean
22	T2	The pharmaceutical faculty has modern equipment (drug delivery screens, payment machines, software)
23	T3	The pharmacist’s outfit is neat, polite, and full of name plates
24	T4	Instruction pictures and tables leading to the dispensing area are easy to understand
25	T5	The medicine dispensing area is fully equipped with room facilities against COVID-19
26	T6	The medicine dispensing room meets the quality standard
27	T7	The waiting area for drug dispensing is spacious and clean
28	T8	Drugs are neatly arranged
**General satisfaction (GS)**		
29	GS	Outpatients are satisfied with the health insurance drug dispensing at Cho Ray Hospital

DISCLAIMER: This is an unofficial translation; therefore no legal rights can be reserved based on this document.

**Table 2:** Characteristics of the outpatients.

Items	Categories	Number (%)
Age	≤24	24 (11.4%)
	25–54	78 (37.2%)
	≥55	108 (51.4%)
Gender	Male	91 (43.3%)
	Female	119 (56.7%)
Level of education	Secondary school or lower	44 (20.9%)
	High school	91 (43.3%)
	College/University	65 (31.0%)
	Post-graduate	10 (4.8%)
Occupation	Officials	54 (25.7%)
	Freelancer	108 (51.4%)
	Retired	9 (4.3%)
	Others	39 (18.6%)

**Table 3:** The questionnaire’s validity and reliability analyses, considering the mean score for patient satisfaction for each domain and its items.

No	Code	Mean	SD	Cronbach’s Alpha	Corrected Item—Total Correlation	Cronbach’s Alpha if Item Deleted	Cronbach’s Alpha	Corrected Item—Total Correlation
Reliability		3.8524	0.61723					
1	REL1	3.5143	0.97445	0.747	0.381	0.747		
2	REL2	3.5348	0.81404		0.596	0.614	0.747	0.546
3	REL3	4.2571	0.74921		0.520	0.659		0.570
4	REL4	3.5033	0.85355		0.577	0.627		0.605
Responsiveness		3.3514	0.54512					
5	RES1	3.6048	0.76463	0.852	0.646	0.826	0.852	0.646
6	RES2	3.3190	0.69041		0.716	0.807		0.716
7	RES3	3.1805	0.81370		0.726	0.804		0.726
8	RES4	3.4619	0.72612		0.647	0.826		0.647
9	RES5	3.1910	0.73571		0.587	0.841		0.587
Assurance		3.3299	0.79992					
10	A1	3.4905	1.04098	0.911	0.644	0.908	0.911	0.644
11	A2	3.2524	1.05273		0.772	0.893		0.772
12	A3	3.4857	0.97935		0.745	0.897		0.745
13	A4	3.1762	1.11209		0.607	0.911		0.607
14	A5	3.3286	1.03139		0.768	0.894		0.768
15	A6	3.4333	1.07953		0.814	0.889		0.814
16	A7	3.1429	1.12329		0.779	0.892		0.779
Sympathy		3.3893	0.70069					
17	DC1	3.4667	0.90788	0.809	0.588	0.778	0.809	0.588
18	DC2	3.4524	0.90724		0.588	0.778		0.588
19	DC3	3.4095	0.98014		0.659	0.744		0.659
20	DC4	3.2286	0.76724		0.669	0.739		0.669
Tangible		3.6899	0.54124					
21	T1	3.3952	0.95395	0.832	0.367	0.843		
22	T2	3.6952	0.77180		0.680	0.797	0.843	0.642
23	T3	3.6190	0.79929		0.617	0.804		0.624
24	T4	3.7476	0.76284		0.629	0.803		0.626
25	T5	3.9810	0.80644		0.591	0.808		0.595
26	T6	3.7714	0.73540		0.569	0.811		0.595
27	T7	3.7190	0.77158		0.607	0.807		0.624
28	T8	3.5905	0.83231		0.461	0.824		0.478
**General satisfaction**		**3.4238**	**0.78862**					

A: assurance; REL: reliability; RES: responsiveness; S: sympathy; T: tangibles.

**Table 4:** Exploratory factor analysis.

Items	Factors				
	A	T	RES	S	REL
A1	0.705				
A2	0.822				
A3	0.802				
A4	0.726				
A5	0.848				
A6	0.856				
A7	0.845				
T2		0.753			
T3		0.714			
T4		0.676			
T5		0.626			
T6		0.697			
T7		0.776			
T8		0.625			
RES1			0.769		
RES2			0.827		
RES3			0.819		
RES4			0.764		
RES5			0.657		
S1				0.736	
S3				0.731	
S2				0.559	
S4				0.773	
REL2					0.736
REL3					0.780
REL4					0.799
KMO			0.847		
*p*-value in the Bartlett test			<0.001		
Total variance explained			62.891		
Eigenvalue			1.262		

A: assurance; REL: reliability; RES: responsiveness; S: sympathy; T: tangibles.

**Table 5:** Correlation coefficient matrix of the factors with general satisfaction.

		REL	RES	A	S	T
GS	Corr	0.415 **	0.235 **	0.130 *	0.285 **	0.151 *
	*p*-value (2-tailed)	<0.001	0.001	0.039	<0.001	0.028

** *p*-value < 0.01 (two-tailed); * *p*-value < 0.05 (two-tailed); A: assurance; GS: general satisfaction; REL: reliability; RES: responsiveness; S: sympathy; T: tangibles.

**Table 6:** Multiple-regression analysis.

Model	Standardized Coefficients	*t*-Test *p*-Value	Multicollinearity	
	Beta		Tolerance	VIF
Intercept		0.743		
REL	0.464	<0.001	0.747	1.339
RES	0.079	0.032	0.746	1.340
A	0.014	0.044	0.572	1.747
S	0.075	0.001	0.519	1.926
T	0.287	0.038	0.635	1.576
R^2^		0.646		
Adjusted-R^2^		0.631		
Durbin—Watson		1.889		
*p*-value ANOVA		<0.001		

A: assurance; REL: reliability; RES: responsiveness; S: sympathy; T: tangibles.

## Data Availability

The datasets generated during the current study are available from the corresponding author upon reasonable request.
